# Alcohol-related dementia – an overlooked entity?

**DOI:** 10.1192/j.eurpsy.2021.1120

**Published:** 2021-08-13

**Authors:** V. Nogueira, M. Mendes, I. Pereira, J. Teixeira

**Affiliations:** Clínica 4 - Unidade De Alcoologia E Novas Dependências, Centro Hospitalar Psiquiátrico de Lisboa, Lisboa, Portugal

**Keywords:** alcohol neurotoxicity, dementia, korsakoff’s syndrome

## Abstract

**Introduction:**

The relationship between alcohol use and dementia is complex. There is a J-shaped relationship between alcohol use and cognitive impairment and evidence shows that one-quarter of the dementia population have alcohol related problems. It is estimated that alcohol-related dementia (ARD) contributes for about 10% of all cases of dementia, especially early-onset dementia, but is largely overlooked or seen as a comorbid factor.

**Objectives:**

To clarify the relationship between alcohol use, alcohol-related brain damage and dementia; to review the clinical features, neuropathology, nosology and neuropsychology of ARD and alcohol-induced persisting amnestic syndrome (Wernicke-Korsakoff syndrome- WKS).

**Methods:**

We performed a review of systematic reviews from the last 10 years. A total of 28 systematic reviews were identified.

**Results:**

Heavy alcohol use has been shown to be a contributory factor and necessary factor in the development of multiple brain diseases. It may cause brain damage in multiple ways: direct neurotoxic effect of acetaldehyde; thiamine deficiency. It is also a risk factor for other conditions, such as hepatic encephalopathy, epilepsy and head injury.

**Conclusions:**

Clinical observation favors the diagnosis of ADR as a distinct entity, but broader evidence reflects significant commonality between ARD and WKS, tough neuropsychological studies have largely attempted to differentiate these syndromes. Repeated episodes of WKS may cause cognitive deterioration. In contrast to other common causes of dementia, the decline in cognitive functioning in ARD is relatively non-progressive if abstinence is maintained, or even partially reversible, as supported by neuroimaging evidence. Given the increase in per capita consumption, it is expected a disproportionate increase in ARD.

